# The Role of Slit-2 in Gestational Diabetes Mellitus and Its Effect on Pregnancy Outcome

**DOI:** 10.3389/fendo.2022.889505

**Published:** 2022-06-23

**Authors:** Yan Wang, Shihua Zhao, Wei Peng, Ying Chen, Jingwei Chi, Kui Che, Yangang Wang

**Affiliations:** ^1^ Department of Endocrinology, The Affiliated Hospital of Qingdao University, Qingdao, China; ^2^ Department of Obstetrics and Gynecology, The Affiliated Hospital of Qingdao University, Qingdao, China; ^3^ Qingdao Key Laboratory of Thyroid Diseases, The Affiliated Hospital of Qingdao University, Qingdao, China

**Keywords:** Slit guidance ligand 2, gestational diabetes mellitus (GDM), cord blood, pregnancy outcome, peripheral blood

## Abstract

**Background:**

Slit guidance ligand 2 (Slit-2), as a member of the Slit family, can regulate the inflammatory response and glucose metabolism. The purpose of this study was to explore the expression of Slit-2 in maternal peripheral blood and neonatal cord blood of gestational diabetes mellitus (GDM) patients and its potential importance in disease progression.

**Methods:**

This study included 57 healthy pregnant women and 61 GDM patients. The levels of Slit-2, C-reactive protein (CRP), monocyte chemoattractant protein-1 (MCP-1), C-peptide (C-P), galectin-3(Gal-3), HbA1c, fasting blood glucose (FBG) and fasting insulin (FINS) in maternal peripheral blood and neonatal cord blood were detected by ELISA. Spearman’s rank correlation test was used to assess the association between peripheral Slit-2 and inflammatory indicators, insulin resistance, and pregnancy outcomes. Logistic regression analysis was used to analyze the risk factors of GDM.

**Results:**

Slit-2 levels in maternal peripheral blood and neonatal cord blood of the GDM patients were higher than those of the HC. Slit-2 levels in maternal peripheral blood and neonatal cord blood of the GDM patients were positively correlated with inflammatory factors CRP and MCP-1 levels. The level of Slit-2 in the maternal peripheral blood of the GDM patients was positively correlated with the level of homeostasis model assessment insulin resistance (HOMA-IR) and HbA1c in maternal peripheral blood, but was negatively correlated with the level of homeostasis model assessment –β (HOMA-β). We also found that the Slit-2 level in the maternal peripheral blood of the GDM patients was negatively correlated with neonatal blood glucose, positively correlated with neonatal weight and independent of neonatal total bilirubin.

**Conclusion:**

Our study suggests that the abnormal increase in Slit-2 in GDM may be related to its pathogenesis, and it was correlated with neonatal blood glucose and weight in patients with GDM, suggesting that Slit-2 may be a potential biomarker of GDM.

## Introduction

Gestational diabetes mellitus (GDM) is a common complication of pregnancy and occurs the first time that blood glucose levels are elevated during pregnancy. The incidence rate of this condition is 9%-25% worldwide (1, 2). GDM is associated with insulin resistance (3, 4), the inflammatory response (5, 6), islet β cell dysfunction and obesity (7). GDM not only increases the risk of metabolic diseases but also leads to adverse pregnancy outcomes such as neonatal hypoglycemia, macrosomia, jaundice and fetal distress (8–11). Therefore, it is important to explore the pathogenesis of GDM and to prevent, monitor and treat GDM in a timely and effective manner.

Slit guidance ligand 2 (Slit-2) is a new type of adipoprotein, and the full-length Slit-2 protein is a secretory ligand. This protein splits into two fragments, a 140 kDa N-terminal product (Slit-2-N) and a 50–60 kDa C-terminal product (Slit-2-C) (12), and interacts with Robo receptors. Robo receptors are divided into Robo1, Robo2, Robo3 and Robo4, and the binding of Slit-2 to specific Robo receptors regulates specific cell functions (13–15). Slit-2 has been reported to play an important role in neuronal and vascular development (16–18). Slit-2 is also involved in the development of many organs and is related to cancer apoptosis, migration, invasion, occurrence and development (19, 20). In addition, Slit-2 can regulate different inflammatory diseases and inflammatory phenotypes and then determine the activity and severity of the disease (21, 22). Recently, the role of Slit-2 in glucose metabolism has become a new research hotspot. Zhou et al. confirmed that Slit-2 concentration in the vitreous fluid of patients with diabetes was significantly higher than that of nondiabetic patients through a diabetic rat model and proposed the role of Slit-Robo signaling in different stages of diabetic retinopathy (23). Svensson et al. proposed that Slit-2, as a beige fat secretion factor (24), has certain influence on adipose tissue homeostasis and glucose metabolism under the control of PRDM16 and cold exposure. Studies have further confirmed that peripheral Slit-2 is related to human serum glucose level and insulin secretion function (25). In addition, it has been proved that Slit-2 overexpression increases the diameter of maternal blood sinuses and fetal capillaries, promoting vascular remodeling in the Slit-2 overexpression mouse model (26). Li et al. found that Slit-2/Robo1 signal could regulate trophoblast differentiation and invasion, thereby limiting β-subunit of human chorionic gonadotropin (β-HCG) production and inhibiting placental angiogenesis, leading to abortion and threatened abortion in early pregnancy (27). Tiensuu H et al. proposed that the risk of spontaneous preterm birth and fetal growth is associated with the level of Slit-2 (28). In short, Slit-2 has certain effects on glucose metabolism and pregnancy outcomes, but the role of Slit-2 in GDM and its pregnancy outcomes remain unclear.

Galectin-3(Gal-3) is a member of galectin family (29), which has the effects of promoting fibrosis and inflammation (30). Many studies have shown that the imbalance of serum Gal-3 level in patients with GDM may be an important predictor of GDM (31–33). Patients with GDM have systemic inflammatory response (34, 35), and the inflammatory factors C-reactive protein (CRP) and monocyte chemoattractant protein-1 (MCP-1) are significantly increased in patients with GDM, which are involved in the occurrence and development of GDM (36, 37). HbA1c represents the level of glycosylated hemoglobin, which can reflect the average blood glucose level in the past 2-3 months, so it is necessary to detect the HbA1c in GDM patients1-2 (38, 39). GDM is also closely related to the increase of homeostasis model assessment insulin resistance (HOMA-IR) and the decrease of homeostasis model assessment-β (HOMA-β) (40, 41), which is also one of the important characteristics of GDM.

Therefore, in this study, we explored the level of Slit-2 in maternal peripheral blood and neonatal cord blood of GDM patients, its relationship with inflammatory factors, insulin resistance, islet β cell function and the correlation with Gal-3. In addition, we explored the relationship between Slit-2 levels in maternal peripheral blood and neonatal cord blood of GDM patients and adverse pregnancy outcomes to further understand the role of peripheral blood Slit-2 in glucose metabolism.

## Methods

### Study Populations

From September 2018 to March 2019, we selected 67 pregnant women with GDM and 66 healthy pregnant women who came to the Affiliated Hospital of Qingdao University for regular routine obstetric examination as the research subjects. GDM was defined according to the Chinese guidelines for the prevention and treatment of diabetes (42). The inclusion criteria were as follows: previous physical health, no history of drug and alcohol abuse, age-appropriate pregnancy (aged 20–40 years), and no other pregnancy complications except gestational diabetes mellitus. Fifteen participants were excluded because of gestational hypertension (six cases), preeclampsia (three cases), acute fatty liver in pregnancy (one case) and premature delivery (five cases). Finally, 61 GDM patients and 57 healthy control (HC) were selected as the research subjects, all subjects were not disturbed by exogenous insulin. All participants were informed and signed a consent form. The experimental protocol was formulated according to the Declaration of Helsinki in 1964 and was consistent with the guidelines of the Human Ethics Committee of the Affiliated Hospital of Qingdao University (QYFYWZLL26496) (43).

### Clinical Data

The height (cm), weight (kg), waist circumference (cm) and blood pressure (mmHg) of all subjects on the day of delivery were measured, and the BMI [weight (kg)/height (m^2^)] was calculated. Sex, birth height (cm), birth weight (kg), blood glucose (mmol/l) and Apgar score of newborns were recorded. The knee joint, hip joint and head of the newborn were fixed, and the height of the newborn was measured from the highest point of the top of the head to the highest point of the foot with tape. The weight of the newborn was measured with a baby scale, the newborn was placed in the center of the scale, and the weight of the newborn was read (kg). After the fingertips of the newborns were disinfected with 75% alcohol, the blood glucose of the newborns was detected by a fingertip blood glucose detector. The Apgar score was calculated according to skin color, heart rate, respiration, muscle tension and reflex.

### Detection of Maternal Peripheral Blood and Neonatal Cord Blood by ELISAs

Fasting blood of pregnant women before delivery and cord blood of newborns were collected on the day of delivery. Neonatal cord blood samples were collected in the umbilical artery within 5 minutes after delivery. The samples were centrifuged twice (3000 rpm/min) in a centrifuge for 10 minutes each time, and the collected serum was stored at -80°C until use. The levels of Slit-2, C-reactive protein (CRP), monocyte chemoattractant protein-1 (MCP-1), C-peptide (C-P), HbA1c, fasting insulin (FINS), fasting blood glucose (FBG) and galectin-3(Gal-3) in maternal peripheral blood and neonatal cord blood were detected by ELISA kits (Yilairuite Biotech Co., Wuhan, China). Three wells were set for all samples, and the average value was taken as the final value.


HOMA−IR=FBG (mmol/L) ×FINS(μmU/mL) / 22.5.

HOMA−β=20×FINS(μmU/mL)/(FBG(mmol/L)−3.5)

### Statistical Analysis

Standard statistical analysis was conducted using GraphPad Prism 8 and SPSS, version 22.0 (IBM, Armonk, NY). Normality of variables was determined through a Shapiro–Wilk test. Qualitative variables are expressed as percentages, and quantitative variables are expressed as the mean ± standard deviation. A t-test was used for intergroup continuous variable comparisons, and a χ^2^ test was used for intergroup categorical variable comparisons. Spearman’s rank correlation test was performed to study the correlation between clinical parameters. A P value of <0.05 was considered statistically Multivariable logistic regression analysis was applied to identify the risk factors of GDM, using the factors with P < 0.05 in the univariable analysis.

## Results

### Clinical and Demographic Characteristics of Subjects

A total of 118 subjects participated in the study: 57 HC and 61 patients with GDM. The weight of newborns in the GDM group (3494.38 ± 459.01 g) was higher than that in the HC group (3307.49 ± 397.53 g), and the blood glucose of the newborns in the GDM group (3.76 ± 1.46 mmol/L) was lower than that in the HC group (4.35 ± 1.46 mmol/L). There was no significant difference in maternal height, blood pressure, gestational age, BMI or gestational weeks between the GDM group and the HC group, and there was no significant difference in neonatal height, sex or Apgar score between the GDM group and the HC group, as shown in **Table 1**. The levels of Slit-2, CRP, MCP-1 HbA1c and FINS in maternal peripheral blood and neonatal cord blood in GDM group were higher than those in HC group, and the level of FBG in neonatal cord blood was lower than that in HC group, as shown in **Table 2**.

**Table 1:** Baseline characteristics of study population.

	Control (n=57)	GDM (n=61)	p value
Maternal age (years)	31.25 ± 5.54	32.49 ± 4.35	0.178
Maternal BMI (kg/m2)	20.33 ± 2.72	21.32 ± 3.24	0.078
Maternal BMI (at birth, (kg/m2))	27.44 ± 5.16	28.76 ± 4.27	0.132
Systolic Blood pressure (mmHg)	113.5 ± 11.2	111.2 ± 10.3	0.255
Diastolic Blood pressure (mmHg)	75.5 ± 8.0	75.3 ± 9.4	0.914
Gestational weeks	39.29 ± 0.82	39.58 ± 1.01	0.102
Vaginal delivery	34 (59.65%)	33 (54.10%)	0.543
Fetal sex (male)	28 (49.12%)	32 (52.45%)	0.717
Birth weight (g)	3307.49 ± 397.53	3494.38 ± 459.01	0.021
Birth height (cm)	50.7 ± 1.4	51.2 ± 1.4	0.122
Blood glucose (mmol/L)	4.35 ± 1.46	3.76 ± 1.46	0.030
Birth Apgar (5min)	9.5 ± 0.5	9.4 ± 0.5	0.899

**Table 2:** Research results of study population.

	Control (n=57)	GDM (n=61)	p value
Maternal Slit-2 (ng/ml)	1.36 ± 0.46	2.66 ± 0.82	0.000
Maternal C-P (mIU/L)	0.91 ± 0.29	1.05 ± 0.39	0.032
Maternal MCP-1 (pg/ml)	187.34 ± 38.77	202.49 ± 41.78	0.044
Maternal CRP (μg/ml)	75.95 ± 21.17	84.65 ± 21.04	0.027
Maternal Galectin-3 (ng/mL)	8.02 ± 0.76	32.08 ± 2.75	0.000
Maternal FINS (mIU/L)	7.88 ± 0.70	15.28 ± 1.28	0.000
Maternal FBG (mmol/L)	4.32 ± 0.39	7.33 ± 1.22	0.000
HbA1c (%)	4.73 ± 0.42	7.50 ± 0.88	0.000
Maternal HOMA-IR	1.51 ± 0.20	4.99 ± 1.00	0.000
Maternal HOMA-β	261.65 ± 182.15	89.78 ± 34.43	0.000
Neonatal Slit-2 (ng/ml)	0.79 ± 0.27	0.97 ± 0.30	0.001
Neonatal MCP-1 (pg/ml)	83.93 ± 14.36	90.97 ± 21.60	0.038
Neonatal CRP (μg/ml)	39.10 ± 12.71	43.82 ± 6.59	0.014
Neonatal Galectin-3 (ng/mL)	1.65 ± 0.42	1.69 ± 0.29	0.560
Neonatal FINS (mIU/L)	8.03 ± 1.39	16.13 ± 2.77	0.000
Neonatal FBG (mmol/L)	3.36 ± 0.87	2.61 ± 0.92	0.000
Neonatal total bilirubin (umol/L)	147.37 ± 23.16	155.34 ± 26.67	0.086
Neonatal HOMA-IR	1.21 ± 0.39	1.86 ± 0.71	0.000

### Increased Levels of Maternal Peripheral Blood and Neonatal Cord Blood Slit-2 in GDM Patients

**Figure 1:**
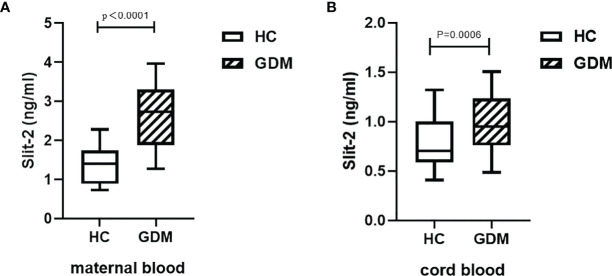
Slit-2 levels in maternal peripheral blood and neonatal cord blood of GDM patients and HC. **(A)** Comparison of Slit-2 levels in maternal peripheral blood between the GDM patients and the HC. **(B)** Comparison of neonatal cord blood Slit-2 levels between the GDM patients and the HC. Slit-2, Slit guidance ligand 2; HC, Healthy Control; GDM, gestational diabetes mellitus.

We investigated the changes in maternal peripheral blood and neonatal cord blood Slit-2 levels between the GDM patients and the HC. The level of maternal peripheral blood Slit-2 in the GDM patients was higher than that in the HC (*P* < 0.0001; **Figure 1A**). The Slit-2 level in the neonatal cord blood of the GDM patients was also higher than that of the HC (*P* < 0.0006; **Figure 1B**).

### Association Between Slit-2 Levels in Maternal Peripheral Blood and Neonatal Cord Blood and Inflammatory Factors in GDM Patients

**Figure 2:**
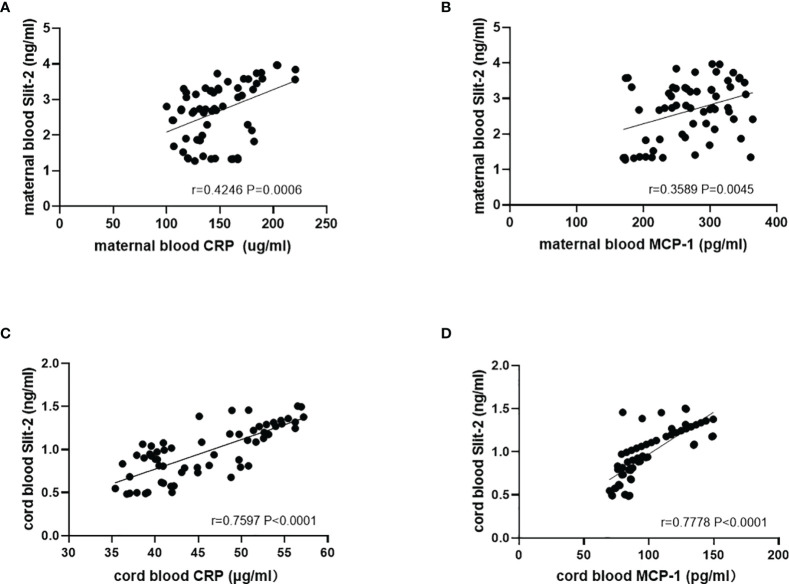
Relationship between the Slit-2 level and CRP and MCP-1 in maternal peripheral blood and neonatal cord blood of patients with GDM. **(A)** Relationship between the Slit-2 level and CRP in maternal peripheral blood of the patients with GDM. **(B)** Relationship between the Slit-2 level and MCP-1 in maternal peripheral blood of the patients with GDM. **(C)** Relationship between the Slit-2 level and CRP in neonatal cord blood of the patients with GDM. **(D)** Relationship between the Slit-2 level and MCP-1 in neonatal cord blood of the patients with GDM. Slit-2, Slit guidance ligand 2; CRP, C-reactive protein; MCP-1, monocyte chemoattractant protein-1.

We analyzed the correlation between Slit-2 levels in maternal peripheral blood and neonatal cord blood and inflammatory factor CRP and MCP-1 levels. The level of Slit-2 in maternal peripheral blood was positively correlated with the CRP and MCP-1 levels (*P*=0.0006, r=0.4246; *P*= 0.0045, r=0.3589; **Figures 2A, B**). The level of Slit-2 in neonatal cord blood was also positively correlated with the inflammatory factors CRP and MCP-1 (*P*<0.0001, r=0.7597; *P*<0.0001, r=0.7778; **Figures 2C, D**).

### Association Between Slit-2, HbA1c and HOMA in Maternal Peripheral Blood and Neonatal Cord Blood in GDM Patients

**Figure 3:**
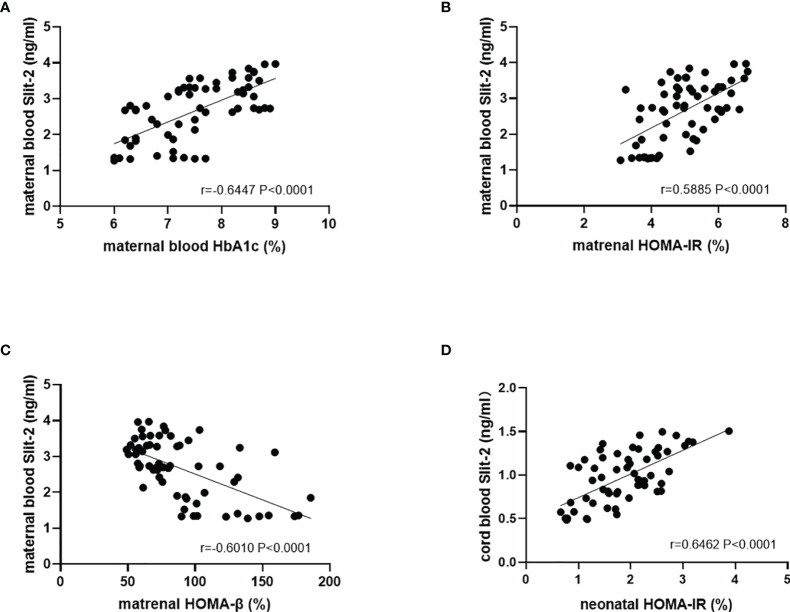
Correlation between the Slit-2, HbA1c and HOMA in maternal peripheral blood and neonatal cord blood of the patients with GDM. **(A)** Correlation between the Slit-2 level and HbA1c in maternal peripheral blood of the patients with GDM. **(B)** Correlation between the Slit-2 level and HOMA-IR in maternal peripheral blood of the patients with GDM. **(C)** Correlation between the Slit-2 level and HOMA-β in maternal peripheral blood of the patients with GDM. **(D)** Correlation between the Slit-2 level and HOMA-IR in neonatal cord blood of the patients with GDM. Slit-2, Slit guidance ligand 2; HOMA-IR, Homeostasis model assessment insulin resistance; HOMA-β, Homeostasis model assessment -β.

We investigated the association between Slit-2 levels in maternal peripheral blood and neonatal cord blood HbA1c and HOMA steady state model to evaluate islet β cell function and Insulin Resistance level. The level of Slit-2 in maternal peripheral blood was positively association with HbA1c and HOMA-IR but negatively association with HOMA-β in maternal peripheral blood. (P<0.0001, r=0.6447 **Figure 3A**; P<0.0001, r=0.5885 **Figure 3B**; P<0.0001, r=-0.6010 **Figure 3C**). The level of Slit-2 in neonatal cord blood was significantly positively correlated with HOMA-IR level in neonatal cord blood (P < 0.0001, r=-0.6462; **Figure 3D**). Due to the immature neonatal islet β cell function, there is no assessment of neonatal cord blood HOMA-β and the correlation with neonatal cord blood Slit-2.

### Association Between Slit-2 Level and Gal-3 Level in Maternal Peripheral Blood and Neonatal Cord Blood in GDM Patients

**Figure 4:**
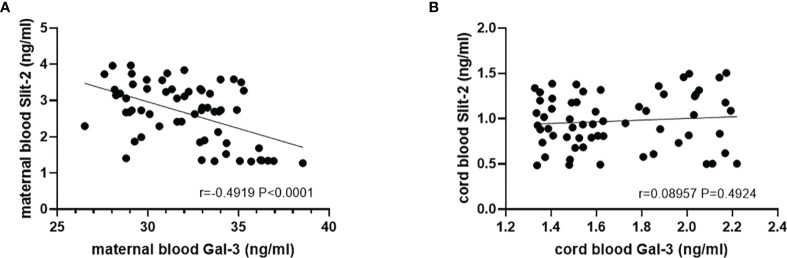
Correlation between the Slit-2 level and Gal-3 level in maternal peripheral blood and neonatal cord blood of the patients with GDM. **(A)** Correlation between the Slit-2 level and Gal-3 level in maternal peripheral blood of the patients with GDM. **(B)** Correlation between the Slit-2 level and Gal-3 level in neonatal cord blood of the patients with GDM. Slit-2 (Slit guidance ligand 2); Gal-3 (Galectin 3).

The Slit-2 levels in maternal peripheral blood were negatively correlated with the Gal-3 level in maternal peripheral blood (*P*<0.0001; r=-0.4919; **Figure 4A**). Correlation analysis showed that the levels of neonatal cord blood Slit-2 had no significant correlation with the Gal-3 level in neonatal cord blood (*P*=0.49224, r=0.08957; **Figure 4B**).

### Association of Maternal Peripheral Blood and Neonatal Cord Blood Slit-2 Expression With Adverse Pregnancy Outcomes in GDM Patients

**Figure 5:**
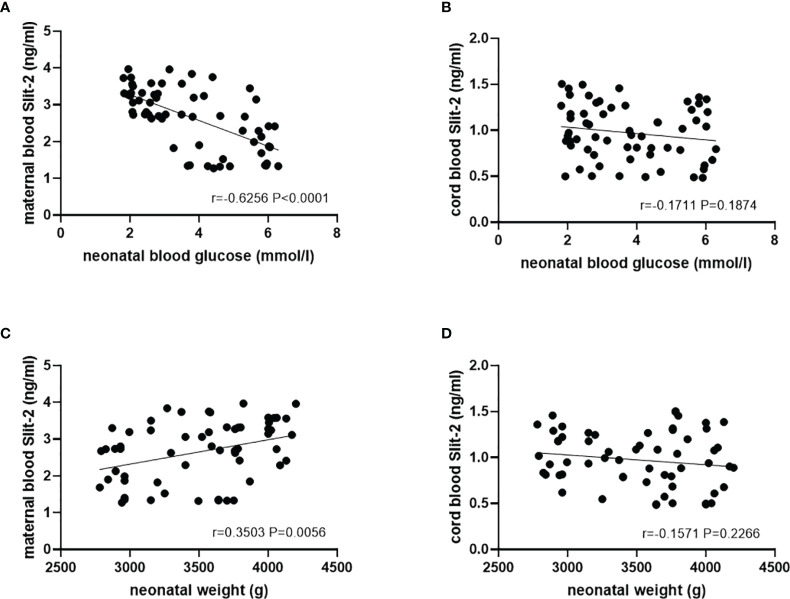
Relationship between the Slit-2 level in maternal peripheral blood and neonatal cord blood and adverse pregnancy outcomes of the patients with GDM. **(A)** Correlation between the Slit-2 level in maternal peripheral blood and neonatal blood glucose of the patients with GDM. **(B)** Correlation between the level of Slit-2 in neonatal cord blood and neonatal blood glucose of the patients with GDM. **(C)** Correlation between the Slit-2 level in maternal peripheral blood and neonatal weight of the patients with GDM. **(D)** Correlation between the level of Slit-2 in neonatal cord blood and neonatal weight of the patients with GDM. Slit-2, Slit guidance ligand 2.

In the patients with GDM, we investigated the association of maternal peripheral blood and neonatal cord blood Slit-2 overexpression with neonatal blood glucose, neonatal weight and neonatal total bilirubin. The results showed that there was a negative correlation between maternal Slit-2 level and neonatal blood glucose (*P*<0.0001, r=-0.6256; **Figure 5A**). The level of Slit-2 in neonatal cord blood was also negatively correlated with neonatal blood glucose, although not significantly (*P* = 0.1874, r =-0.1711; **Figure 5B**). The level of Slit-2 in maternal peripheral blood was positively correlated with the weight of newborns (*P*=0.0056, r=0.3503; **Figure 5C**) and that of neonatal cord blood was not related to the weight of newborns (*P*=0.2266, r=0.1571; **Figure 5D**). The level of Slit-2 in maternal peripheral blood was not related to neonatal total bilirubin (P=0.5777, r=0.07269).

### Risk Factors of GDM Patients Were Detected by Logistic Regression

We evaluated the risk factors of GDM patients. In univariate analysis, Slit-2, C-P, CRP, MCP-1 and Gal-3 in peripheral blood of GDM patients were the risk factors of GDM patients. After adjustment of multivariate logistic regression analysis, it is confirmed that the levels of Slit-2 and Gal-3 in maternal peripheral blood are risk factors for GDM patients (**Table 3**).

**Table 3:** Logistic regression analysis for GDM.

	Univariable			Multivariable		
	Hazard Ratio	95% CI	P value	Hazard Ratio	95% CI	P value
Maternal age (years)	1.052	0.977-1.132	0.177			
Maternal BMI (kg/m2)	1.115	0.987-1.260	0.079			
Maternal BMI (at birth, (kg/m2))	1.061	0.982-1.146	0.134			
Gestational weeks	1.391	0.936-2.069	0.103			
Maternal Slit-2 (ng/ml)	14.159	5.573-35.976	0.000	18.789	6.227-56.691	0.000
Maternal C-P (mIU/L)	3.139	1.083-9.101	0.035	2.377	0.510-11.087	0.270
Maternal MCP-1 (pg/ml)	1.009	1.000-1.019	0.046	0.990	0.976-1.005	0.186
Maternal CRP (μg/ml)	1.020	1.002-1.038	0.030	1.005	0.963-1.050	0.815
Maternal Galectin-3 (ng/mL)	3.560	2.306-5.479	0.000	5.612	2.417-13.027	0.000

## Discussion

In this study, we elucidated the correlation between peripheral Slit-2 and GDM patients and newborns for the first time. Maternal and fetal material exchange through the placenta. Maternal blood first contact with the placenta, then the umbilical vein to the fetus. Fetal blood contacts with umbilical artery first and then passes through the placenta to maternal blood (44). Slit-2, CRP, MCP-1, Gal-3, FINS and other indexes in blood complete maternal-fetal blood circulation through placenta transmission (28, 44–51). Therefore, we measured maternal peripheral blood and neonatal umbilical artery blood to reflect the metabolic concentration of maternal and neonatal. Some important findings emerge out of the present study, Slit-2 levels in maternal peripheral blood and neonatal cord blood of the GDM patients were significantly increased and were positively correlated with inflammatory factors, including CRP and MCP-1 levels. In addition, the level of Slit-2 in maternal peripheral blood was positively correlated with HbA1c and HOMA-IR but negatively correlated with HOMA-β in maternal peripheral blood. The Slit-2 level in the neonatal cord blood of the GDM patients was positively correlated with the HOMA-IR level in neonatal cord blood, and the Slit-2 level in the maternal peripheral blood of the GDM patients was negatively correlated with the Gal-3 level in maternal peripheral blood. The study also demonstrated that the Slit-2 level in maternal peripheral blood was negatively correlated with neonatal glycemia, positively correlated with neonatal weight. Moreover, we proved that Slit-2 may be a risk factor for patients with GDM by logistic regression analysis.

Slit-2, a secreted extracellular matrix protein, is a homologous protein of Slit (21). In recent years, the role of Slit-2 in glucose metabolism has attracted much attention. Studies have shown that Slit-2 is expressed in the fibrous vascular membrane of diabetic patients, and Slit-2/Robo1 signaling has been proved to contribute to the development of diabetic retinopathy (52). Slit-2/Robo1 signaling is involved in early diabetic nephropathy and may be an effective therapeutic target for abnormal angiogenesis in early diabetic nephropathy (53). Slit-2/Robo4 plays an important role in the occurrence and development of Type 1 Diabetes Mellitus (54). In addition, Kang et al. investigated Slit-2 levels in human serum and determined the role of Slit-2 in diabetes (25). In our study, we assessed the levels of Slit-2 in maternal and cord blood in HC and GDM patients and discovered that Slit-2 was significantly increased in maternal peripheral blood and neonatal cord blood in GDM patients. Moreover, we proved that Slit-2 may be a risk factor for GDM patients through logistic regression analysis. In addition, we also found that the level of Slit-2 in maternal peripheral blood was positively correlated with HOMA-IR and negatively correlated with HOMA-β. The results were consistent with Kang et al.’s study on peripheral Slit-2 and HOMA-β in diabetic patients (25). Yang et al. confirmed that Slit-2 is expressed in islet β cells and Slit/Robo signal regulates the survival of β cells by regulating apoptosis (55). HOMA-IR as an indicator of insulin resistance and HOMA-β as an indicator of islet β cell function are correlated with peripheral Slit-2, which may be related to insulin resistance and islet β cell function in GDM patients.

Slit-2 is a double-edged sword in inflammation. Slit-2 has been reported to play an anti-inflammatory role through its specific receptor Robo4, and Slit-2 can also play a proinflammatory role through its other specific receptor Robo1 (15). Chen et al. believed that Slit-2 could indirectly affect the placental microenvironment by regulating the activity and movement of inflammatory macrophages in the placenta (56). In an article on thyroid-associated ophthalmopathy, some scholars proposed that Slit-2 determines the activity and severity of the disease by regulating the inflammatory phenotype of CD34+ orbital fibroblasts (OF) (21). In addition, Slit-2 is overexpressed in periodontitis and aggravates the inflammatory response, lymphocyte/macrophage infiltration and disease progression (22). In brief, Slit-2 is a regulator of inflammatory response. Inflammation plays a central role in GDM, patients with GDM had low-grade inflammatory reaction, and CRP and MCP-1 were increased. In our study on the relationship between Slit-2 and inflammatory factors CRP and MCP-1 in GDM patients, we found that Slit-2 was positively correlated with CRP and MCP-1 in maternal peripheral blood and neonatal cord blood of GDM patients, suggesting that it may play a proinflammatory role in GDM through Slit 2/Robo1 axis (15).

Studies have shown that Gal-3 is involved in the development of prediabetes and diabetes, which may be related to inflammation, insulin resistance and diabetes β cell dysfunction (57). Our study showed that a negative correlation was found between Slit-2 level and Gal-3 level in maternal peripheral blood of GDM patients. Nancy Freitag suggested that the dysregulation of Gal-3 during pregnancy may lead to the effect of the chimera-type lectin to this adverse pregnancy outcome (31). Therefore, we speculated that Slit-2 may affect the progression of GDM and pregnancy outcome by affecting the level of Gal-3.

Slit-2 plays an important role in the placental microenvironment by participating in macrophage migration through the Robo receptor signaling pathway (56). Li et al. speculated that Slit-2/Robo1 signaling may be involved in the pathogenesis of adverse pregnancy outcomes (27). Slit-2/Robo1 signaling regulates cytotrophoblast epithelial-mesenchymal transition (EMT) by affecting the expression of E-cadherin, which eventually leads to superficial trophoblast invasion, missed abortion and threatened abortion (27). In view of the above relationship between Slit-2 and adverse pregnancy outcomes (28), we studied the correlation between Slit-2 and neonatal weight, neonatal blood glucose and neonatal total bilirubin in the GDM patients. Cord artery blood can well reflect the metabolic concentration of infants and can be used to respond to adverse pregnancy outcomes (58, 59). The results showed that Slit-2 in the maternal peripheral blood of the GDM patients was negatively correlated with neonatal blood glucose and positively correlated with neonatal weight, this may increase the incidence of neonatal hypoglycemia and macrosomia, it is consistent with previous research results (56). In this study, we did not evaluate the correlation between Slit-2 and GDM in placental tissues. It has been reported that the expression of Slit-2 was detected in placenta (45, 46), and the effect of maternal obesity on the expression of Slit-2 was also confirmed (51). Tiensuu H et al. proposed that Slit-2/Robo1 signal may be involved in the pathogenesis of adverse pregnancy outcomes through its effect on trophoblast cell function (28). We speculated that Slit-2 in placenta and blood may play a synergistic role in the development of GDM and adverse pregnancy outcomes, which requires further experimental evidence.

Our study has some limitations. First of all, the role of Slit-2 in human peripheral blood was analyzed through a cross-sectional study. Therefore, only relationship of Slit-2 and other clinical parameters could be provided, and no causal relationship could be drawn from the data in this study. Secondly, the sample size of our study population was limited, subgroup analysis and stratified analysis were not performed. In the follow-up study, the sample size should be expanded to verify the specific effects of Slit-2 *in vivo* and *in vitro* in GDM.

In conclusion, we found elevated Slit-2 levels in maternal peripheral blood and neonatal cord blood of GDM patients for the first time. The Slit-2 levels were correlated with HbA1c, inflammatory factors, insulin resistance, islets β Cell function and Gal-3 level. In addition, Slit-2 was also associated with neonatal blood glucose and neonatal weight. Moreover, we proved that Slit-2 may be a risk factor for GDM patients through logistic regression analysis. We speculated that Slit-2 is closely related to the pathogenesis of GDM and may be a key risk factor in the occurrence and development of GDM, which not only provides a theoretical basis for the study of insulin resistance and inflammatory response induced GDM, but also provides a new target for the prevention and treatment of GDM.

## Data Availability Statement

The raw data supporting the conclusions of this article will be made available by the authors, without undue reservation.

## Ethics Statement

The studies involving human participants were reviewed and approved by QYFYWZLL26496. Written informed consent to participate in this study was provided by the participants’ legal guardian/next of kin. Written informed consent was obtained from the individual(s), and minor(s)’ legal guardian/next of kin, for the publication of any potentially identifiable images or data included in this article.

## Author Contribution

Our manuscript has 7 authors, all of whom contributed significantly to this study. Conceived and designed the experiments: YW, SZ, and YGW. Collected the specimen: YW and WP. Conduct experiments: YW, JC, and KC. Analyzed the data: YW and YC. Wrote the paper: YW and SZ. Supervised the paper: YC and YGW. All authors contributed to the article and approved the submitted version.

## Funding

This study received financial support from National Natural Science Foundation of China (grant/award number: 81600601).

## Conflict of Interest

The authors declare that the research was conducted in the absence of any commercial or financial relationships that could be construed as a potential conflict of interest.

## Publisher’s Note

All claims expressed in this article are solely those of the authors and do not necessarily represent those of their affiliated organizations, or those of the publisher, the editors and the reviewers. Any product that may be evaluated in this article, or claim that may be made by its manufacturer, is not guaranteed or endorsed by the publisher.
